# Mineralogical Characterization of Historic Copper Slag to Guide the Recovery of Valuable Metals: A Namibian Case Study

**DOI:** 10.3390/ma16186126

**Published:** 2023-09-08

**Authors:** Godfrey Dzinomwa, Benjamin Mapani, Titus Nghipulile, Kasonde Maweja, Jaquiline Tatenda Kurasha, Martha Amwaama, Kayini Chigayo

**Affiliations:** 1 Department of Civil, Mining, and Process Engineering, Faculty of Engineering and the Built Environment, Namibia University of Science and Technology, Private Bag, Windhoek 13388, Namibia; bmapani@nust.na (B.M.); mkasonde@nust.na (K.M.); jkurasha@nust.na (J.T.K.); mamwaama@nust.na (M.A.); kchigayo@nust.na (K.C.); 2 Minerals Processing Division, Mintek, Private Bag X3015, Randburg 2125, South Africa; titusn@mintek.co.za

**Keywords:** copper slag, mineralogy, slag re-processing, pyrometallurgy, hydrometallurgy

## Abstract

The depletion of the ore reserves in the world necessitates the search for secondary sources such as waste products (tailings and slag). The treatment and cleaning up of such secondary sources also has a positive impact on the environment. A smelter in Namibia we examined had historic slag which accumulated over decades of its operating life, thus posing the challenge of how best to collect representative samples to evaluate and propose viable methods of recovering contained metals. In this study, analytical and mineralogical characterization of the slag was performed using X-ray fluorescence (XRF) analysis, atomic absorption spectrometer (AAS), ICP-OES, scanning electron microscopy energy dispersive spectroscopy (SEM-EDS) analysis, and optical microscopy analysis. The chemical analyses showed that the metal values contained in the slag were mainly copper, lead, and zinc whose average contents were approximately 0.35% Cu, 3% Pb, and 5.5% Zn. About 10.5% Fe was also contained in the slag. Germanium was detected by scanning electron microscopy, but was however below detection limits of the chemical analysis equipment used. Based on the results, approximate conditions under which the different slag phases were formed were estimated and the recovery routes for the various metals were proposed. Analysis by both optical and scanning electron microscopy revealed that Zn and Fe occurred mainly in association with O as oxides, while Cu and Pb were mainly associated with S as sulphides. The slag consisted of three different phases, namely the silicate phase (slag), metallic phase and the sulphide phases. The phases in the slag were mainly silicate phases as well as metallic and sulphide phases. It was observed that the metallic and sulphide phases were dominant in the finer size fractions (−75 µm) whereas the sulphide phase was also present in the coarser size fractions (+300 µm). An important finding from the microscopy examination was that the sulphide phases were interstitial and could be liberated from the slag. This finding meant that liberation and subsequent concentration of the sulphide phases was feasible using conventional processing techniques.

## 1. Introduction

Ore reserves around the world are currently being depleted, with very few new discoveries occurring. This necessitates the search for the secondary sources of metals such as tailings [1,2,3,4], slag [5,6,7,8,9], and electronic waste printed circuit boards [10,11,12,13,14,15,16]. Copper is one of the metals whose ore reserves have been depleted in many areas, with slag re-processing being common as a way of supplementing the fresh concentrates in smelters [17]. Approximately 2 tons of slag per tonne of copper produced is generated in a typical pyrometallurgical process [6,7,18]. Environmental and socio-economic impacts of such slags are some compelling considerations in favour of further recovery of valuable metals [19,20]. The composition of the slag is dependent on the mineralogy of the ore, but it typically contains oxides of gangue elements such as iron, silicon, magnesium, calcium, phosphorus, and copper [21,22,23]. The slag also contains some proportions of copper matte that is mechanically dragged when tapping from the settling zone of the smelter crucible [24].

Valuable metals such as gold, silver, germanium, and copper contained in sulphide concentrates are mainly dissolved in the copper matte phase. However, the operation of the smelters leads to incomplete settling of the molten metals and sulphides from the liquid slag. Maweja et al. [22] have shown that more than 80% of copper in the matte smelting slags was in sulphide form, which is inherent to mechanical drawing of the matte during slag tapping. Converter slags have high copper contents and are not disposed to waste, therefore they are not considered in this study. Table 1 gives the compositions of some copper slags obtained in plants using different processes and equipment. Typical copper slag composition ranges are Fe (as FeO and Fe_3_O_4_) 20–40%; SiO_2_ 25–40%; Al_2_O_3_ up to 10% and CaO up to 10%. 

The variety of slag compositions of Table 1, is inherent to both the concentrate composition and the extraction method applied. Each slag composition requires an appropriate treatment method for further metal recovery or for utilization as construction material [27,28]. Thermal reduction of copper smelter slags with solid carbon is commonly applied in industry to recover valuable metals [21,29,30,31]. This method requires temperatures above the melting temperature of slags, which is 1300–1500 °C. For example, the ST plant in the Democratic Republic of Congo processes 22,000 tons/month of matte smelter slag by direct reduction with coal in a DC current furnace to recover copper, cobalt, zinc, and germanium from a white alloy. The furnace operates at about 1400 °C. Above this temperature, silica reduction becomes significant and this can result in low grade white alloy product. In Turkey, high-pressure oxidative leaching is rather considered for the simultaneous recovery of 90% of copper, cobalt and zinc from Küre-Kastamonu historical slag containing 0.84% Cu, 0.34% Co, 0.23% Zn, 1.3% S, and about 56% (FeO + Fe_3_O_4_) as shown in the work by [32,33] have demonstrated the effects of heat removal (cooling rate) during slag casting and post casting heat treatment on the leaching of copper, cobalt, and zinc from the slag in acidic media. The formation of silica gels in acidic leaching of amorphous slags will hinder separation processes such as solvent extraction of metal ions from the leaching solutions. Bioleaching treatment of smelter slags has been investigated, but the yields of this method remains moderate for copper, zinc and nickel, with 62%, 35%, and 44% dissolution, respectively, after 29 days [34]. The authors have proceeded by further precipitation of the metals under controlled pH conditions. Flotation is extensively used to recover copper and other metals from smelter slags [35,36]. Flotation with xanthate collectors enhanced by chemical and mechanical activation is applied to recover copper from slags with high efficiency above 90% [37]. A comprehensive review of the methods of copper recovery from slags is presented by Kundu et al. [38], where the authors compare the efficiency and environmental impact of methods of flotation, hydrometallurgy and pyrometallurgy to recover copper from the slags. The variety of methods used in industry and the extensive investigation in new processes show the importance of slag characterization as it enables the selection of a technically and economically viable combination of unit operations for the recovery of metals from slags.

Namibia Custom Smelters in the Republic of Namibia is a standalone operation with no mining activities on site (i.e., they buy the concentrates from producers). There is always a continuous need to supplement the primary source with secondary materials such as the smelter slag. Their smelter can treat complex copper concentrates such as those containing arsenic (As). The smelter consists of an Ausmelt top submerged lance furnace, two Peirce Smith converters, a sulphuric acid plant, a slag milling and flotation plant, and an arsenic trioxide production plant. That process allows the smelter to produce blister copper, sulphuric acid and arsenic trioxide. The blister copper (98.5% Cu) is delivered to refineries in Asia and Europe where it is further refined to copper metal. The Arsenic trioxide is sold worldwide, and the sulphuric acid is sold to Namibian mining companies in the uranium and copper industry for leaching purposes. Since the 1900s when the smelter commenced with a blast furnace operation, historic slag has been stockpiled. Acknowledging the improvement in the technology over the decades, the historic slag can possibly be reprocessed to recover copper and other metal values. Previous studies on the slag samples collected on the dumps in the smelter area showed that there were some recoverable metal values including the high-tech elements such as germanium [39,40]. The objective of this study was to characterize that historic slag chemically and mineralogically, explain the conditions under which the phases were formed, and propose the processing routes for various commercially extractable metals. 

## 2. Materials and Methods

The slag heaps at the smelter were in two categories; the pre-1964 slag that accumulated from 1901 to 1964 from the Pb-blast furnace; and the slag that was produced with a relatively new smelter that was installed in 1964. It was deemed that the historic pre-1964 slag heap was more likely to contain higher amounts of valuable metals due to the less developed extractive technology used at the time. The slag heap typically measured 3–5 m in height and about 5 hectares in surface area. A total of 84 holes were drilled on the historic slag heap. Each drill hole was sampled at 1 m intervals with a 2 kg sample being collected. Each sample was logged and described. Similar samples were then composited in groups of 7–10 samples from 12–20 drillholes. These samples were homogenized in groups of four (4), where each sample represented between 12 and 20 drillholes, depending on the log sheet similarity. The four samples weighed about 20–24 kg. The four samples were each conned and quartered, from which a representative sample of 20 kg was obtained. The representative sample ensured that the materials from all depth sections of the slag profile were represented and sampled. The composited sample was then crushed to −5 mm size so as to reduce the grain size variation for analytical purposes.

**Figure 1:**
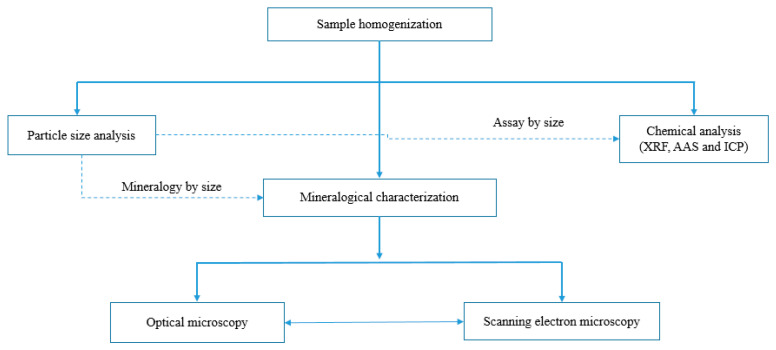
Process diagram for the methodology employed to characterize the copper slag.

The representative 20 kg sample was further blended using the coning and quartering method prior to splitting. The rotary splitter was used to split the blended sample into 1 kg aliquots that were used for particle size analysis, chemical analyses, and mineralogical characterization as shown in Figure 1. 

Particle size analysis was performed on two of the 1 kg sub-samples using different techniques (i.e., one sample was subjected to dry screening using the vibrating sieve shaker for 15 min while the other sample was manually wet screened). Sieves with the aperture sizes ranging between 38 and 3350 µm were used. Wet screening products were dried overnight in the oven set at a temperature of 80 °C. The size-by-size products were reserved for chemical analysis and mineralogical characterization.

Chemical analysis was determined using three different techniques and thus providing validation of the assays. AAS analysis of Fe, Pb, Ca, Zn, and Cu were performed on a Thermo Scientific ICE 3000 series Atomic Absorption Spectrometer (Basel, Switzerland). The ICP-OES analyses were conducted using the Perkin Elmer Optima 8000 (Johannesburg, South Africa). XRF analyses were performed using the benchtop X-ray fluorescence (XRF) spectrometer model NEX CG supplied by Applied Rigaku Technologies from Austin, TX, USA. This benchtop XRF machine uses an in-built calibration procedure referred to as the multi-channel analyser (MCA) Analysis was carried out on a composite head sample as well as samples in the five particle size ranges +3350 µm, +850, +300, +75, and −75 µm. The analysis of the classified slag materials was aimed at identifying the possibility of a preferential partition or concentration of valuable metals in slag particles in certain granulometric sizes. The AAS and ICP-OES samples were digested using aqua-regia (hydrochloric acid: nitric acid [1:3]) solution. For each sample, 1.0 g was added to 25 mL of aqua regia solution in a 100 mL beaker and heated for 2 h. Following digestion, the sample was filtered and transferred with the help of 2% nitric acid in a 100 mL volumetric flask. Appropriate dilution factors were applied to allow for measurements of the concentration of the analytes. 

The microstructures and phase distributions of the granulometric ranges were analyzed in backscattered and in secondary electron modes in a scanning electron microscope (SEM) machine. The samples for microscopy consisted of two types:

1. Loose powders for observation of the particle morphologies and phases in the outer layers of as received condition.
2. Slag material mounted on resin, ground on grit papers then polished to metallographic surface condition using diamond slurries up to the 3 μm for observation inside the cross sections of the particles.

The samples of the materials obtained after granulometric separation in six particle size ranges of +3350, −3350 + 850, −850 + 300, −300 + 75, −75 + 38, and −38 µm were hot mounted on resin and polished for observation using the JEOL JSM-IT300 scanning electron microscope coupled with the Thermo Scientific NORAN System 7 (NS7) energy dispersive spectroscopy (EDS) software (Advancedlab, Basel, Switzerland). The samples were lightly carbon coated using the Quorum Q150T sputter coater (Advancedlab, Switzerland). SEM analysis was carried out in a high vacuum in the backscattered mode (BSE) at an acceleration voltage of 10–15 kV, a probe current of 50 nA, and a working distance of 15 mm. Other samples taken from these classified granulometric size ranges were attached on carbon tapes, lightly carbon coated and observed in the SEM secondary electron (SE) mode without polishing. These samples were also mounted on a polished thin section to allow for observation in a BX51 Olympus optical microscope in both reflection and transmission modes for phase identification.

## 3. Results and Discussion

### 3.1. Particle Size Analysis of the Received Slag Material

**Figure 2:**
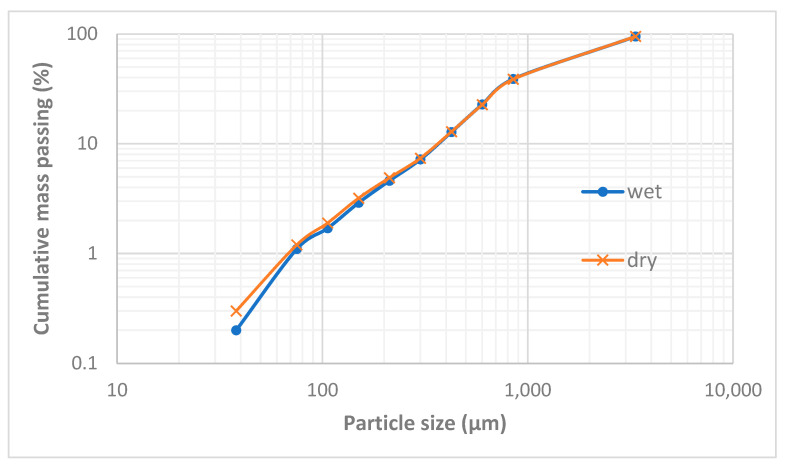
Particle size distribution of unwashed (dry sieved) and water washed (wet sieved) historic smelter slag materials.

Figure 2 presents the particle size distributions of the received smelter slag after wet and dry screening. Since the size distributions of wet and dry sieving matched, it can be concluded that the slag material was not contaminated with soluble materials or the fine airborne materials from the surrounding fields of the dump. It can be observed that the ‘as received’ slag sample was 95% passing 3.35 mm while the particles that are coarser than 300 µm and those finer than 75 µm constitute 92% (*w*/*w*) and 1.2% (*w*/*w*), respectively, of the slag sample. The 80% passing size of the as-received smelter slag material is 2.5 mm. 

### 3.2. Chemical Analysis

The chemical compositions of the composite smelter slag samples are listed in Table 2. The copper assays range between 0.3 and 0.4% in the composite sample while that of iron ranges between 9 and 12%. The assay-by-size (ABS) results are also listed in Table 2 to show the metal distributions in different particle size fraction. It is acknowledged that in Table 2, only the results of the coarsest size fraction (+3.35 mm) and finest size fraction (−75 μm) are presented, with the rest of the ABS results included in Appendix A. In general, it can be observed that the contents of metal values (with the exception of Fe, Zn, and Mn) are higher in the −75 μm (liberated particles) and lowest in the +3.35 mm (unliberated size fraction). Since −75 µm constitutes 1.2% (*w*/*w*) as shown in Figure 2, the assay of 1.2% Cu suggests that about 5% of the total copper in the slag is contained in the −75 µm size fraction while the 95% of the total copper content is still locked in the slag particles coarser than +75 μm. The slag contents in copper and zinc are comparable to those of the DC electric furnace smelter in the Democratic Republic of Congo. Maweja et al. [33] reported the effects of annealing and leaching conditions on the recovery of copper and zinc from the DC electric furnace slag. 

### 3.3. Morphology and Phase Distribution Characterisation of the Slag Materials

#### 3.3.1. Scanning Electron Microscopy

**Figure 3:**
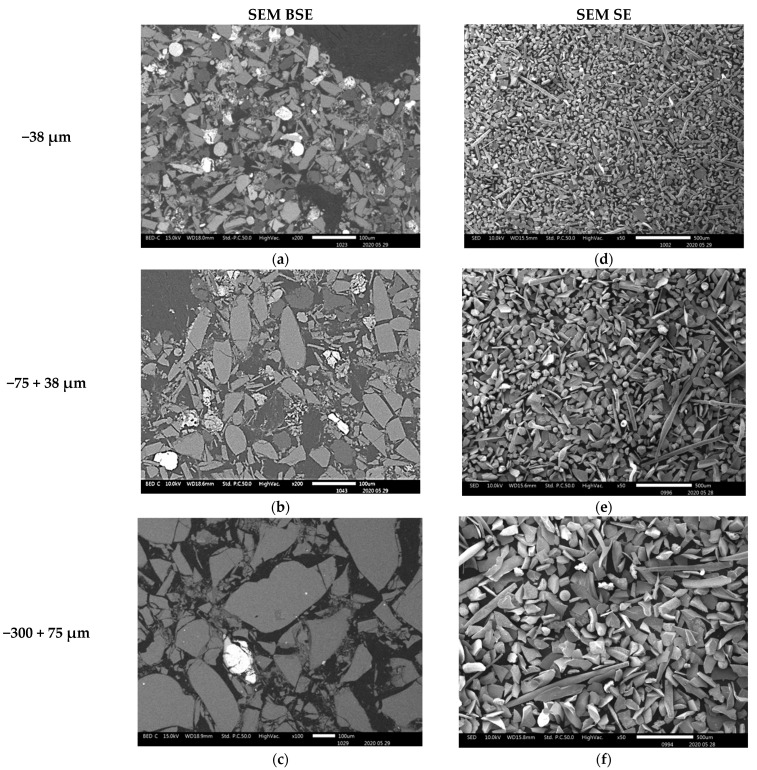
SEM BSE of polished slag particles (**a**–**c**) and SE of received slag material (**d**–**f**) micrographs showing the shapes of the particles. The high reflectance phases are metallic sulphides, whereas the rest of the material are silicates.

**Figure 4:**
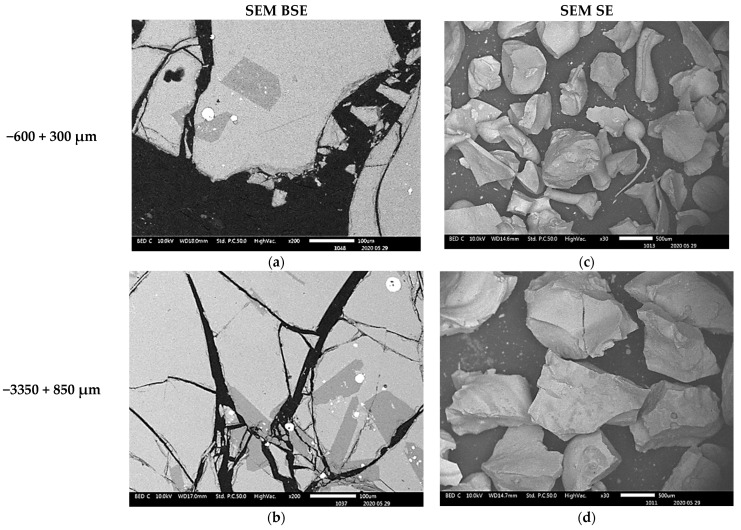
SEM BSE of polished slag particles (**a**,**b**) and SE of received slag material (**c**,**d**) micrographs showing the shapes of the particles. The high reflectance phases are metal sulphides of Cu, Zn, Pb and Fe.

**Figure 5:**
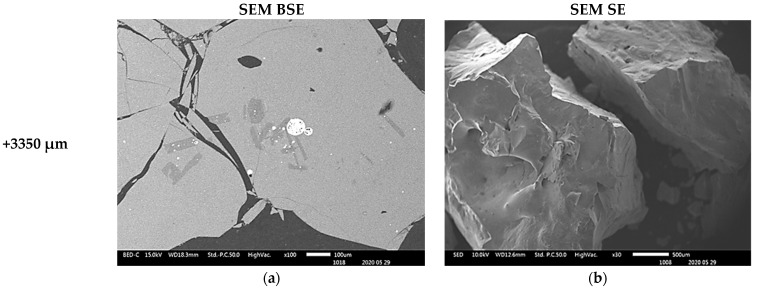
SEM BSE of polished slag particles (**a**) and SE of received slag material (**b**) micrographs showing the shapes of the particles. The high reflectance phases are metal sulphides of Cu, Zn, Pb and Fe.

The compositional contrast observed in backscattered (BSE) mode images (Figure 3a–c, Figure 4a,b and Figure 5a) indicate that higher proportions of particles of material phases containing the heavy elements were found in the fine particles (−38 µm and −75 + 38 µm). The proportion of the phases of heavy elements is lower in the particles coarser than 300 µm. SEM BSE also revealed that the particles of the heavy metal phases present in the fine granulometric size ranges were separated from the slag matrix particles (i.e., higher degree of liberation between particles of metallic and slag phases). The separation of these particles from the slag matrix is attributed to the non-miscibility between the molten slag and the metal compounds contained in the matte that were mechanically dragged when tapping the slag from the hearth zone of the smelting furnace. 

The morphologies and sizes of the particles of the matte or metal phases and the slag matrix particles were investigated under secondary electrons (SE) mode observation in the SEM machine. The images of Figure 3, Figure 4 and Figure 5 illustrate the actual shapes. The SEM SE images indicate higher proportion of the high aspect ratio (Length/Diameter) needle-like particles present in the finer materials of −38 µm (Figure 3c). These needle-like particles are relatively coarser within the fine size material fraction, but their relative volumetric proportion in the slag material decreases as the granulometric size ranges increase in the −75 + 38 µm (Figure 3d) and then in the −300 + 75 µm (Figure 3f). Finally, no needle-like particles are observed in the coarse size ranges of slag material fractions above 300 µm (Figure 4c,d and Figure 5b). The nature and formation mechanism of the needle-like particles is discussed in the next section. 

The scanning electron microscopy images show that the small particles of matte or metal phases (high reflectance) found in the coarse granulometric ranges, above 300 µm (Figure 4 and Figure 5), were rather trapped by occlusion inside the smelting liquid slag and solidified upon quenching in the high pressure water jet. Large slag particles have cracks that formed during the fast quenching in the water jet. The formation of the cracks is attributed to the low thermal conductivity of the slag [41,42,43,44] and the difference in coefficients of thermal expansion, thus shrinkage, between the slag and the matte (metal) phases, which result in thermal stresses inside the solidified material. The particle size distribution in Figure 2 suggested that the materials of granulometric size larger than 300 µm represent about 90% (*w*/*w*) of the sample of the received historic slag material. It, therefore, implies that the major quantities of matte and metal phases, that were mechanically dragged with the slag, are trapped inside the slag particles larger than 300 µm but their concentration values in these large particles are lower than in the finer materials (<300 µm). The fine particle size ranges (<300 μm) represent only about 8% (*w*/*w*) of the historic smelter slag but with higher contents of matte and metal phases. 

#### 3.3.2. Morphology and Composition of Fine Particle Materials

**Figure 6:**
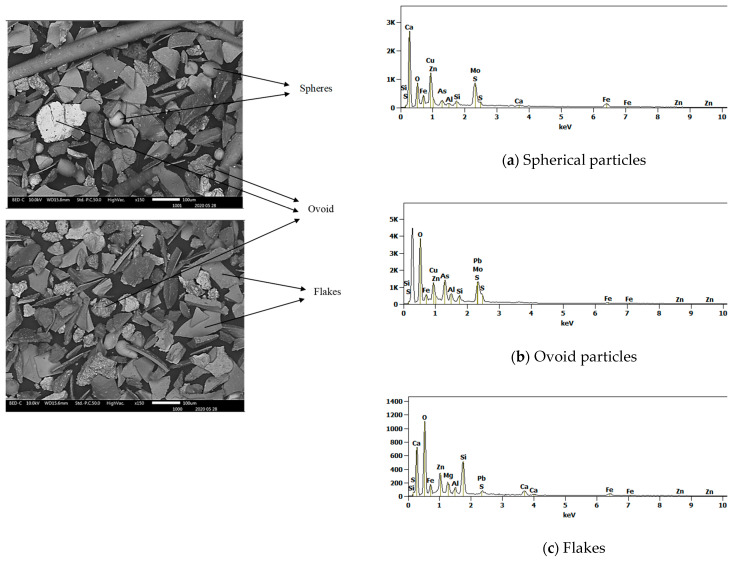
SEM SE images showing particles of different morphologies and sizes in the slag material of granulometric range between 75 and 300 µm and SEM EDS spectra (**a**) spherical particle, (**b**) ovoid particle and (**c**) flakes.

**Figure 7:**
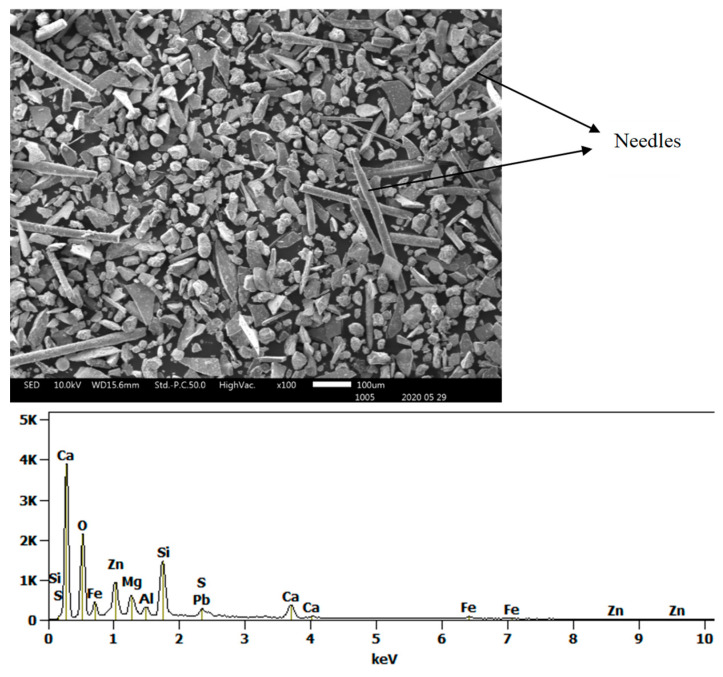
SEM SE image and EDS spectrum showing needle-like slag particles in material received with particle sizes smaller than 38 µm.

The SEM SE observation of particles of the received slag sample material collected in granulometric ranges smaller than 300 µm revealed four types of particle shapes as shown in Figure 6 and Figure 7 (i.e., the flakes, the spheres with smooth surfaces, the needle-like particles, and the ovoid particles with rough surfaces). The SEM SE images of Figure 6 and Figure 7, infer that the flaky particles represent the main constituent of the slag materials in all particle size ranges obtained after sieving the historic slag material. The ovoid particles with rough surfaces are present in a wide size range from 30 to 200 µm. The SEM SE images suggest the ovoid particles are the second important phase present in the material passing the 300 µm sieves. The needle-like particles have aspect ratios (Length/Diameter) higher than 10. Such particles of length bigger than 500 µm have passed through the 38 µm sieve square apertures since their diameter is as small as 25–30 µm. The spherical particles compose the least proportion of phase found in the slag material that passed through the 300 µm sieves. They have a diameter range of 30–70 µm and are distinguishable from the ovoid particles by their smooth surface appearance under SEM SE mode.

Energy dispersive X-ray spectroscopy (EDS) analysis of these four types of particles in the slag sample indicates that they are different phases in compositions. Their corresponding composition ranges are given in Table 3. The EDX method is considered as semi-quantitative, but has the advantage of evaluating the composition of selected grains or regions within the sample of materials under consideration. The method is not recommended for the quantification of light elements, and thus the oxygen contents only serve as qualitative indicators of the oxide phases present in the slag material. Similarly, the sulphur contents reveal the presence of the sulphide phases. 

**Figure 8:**
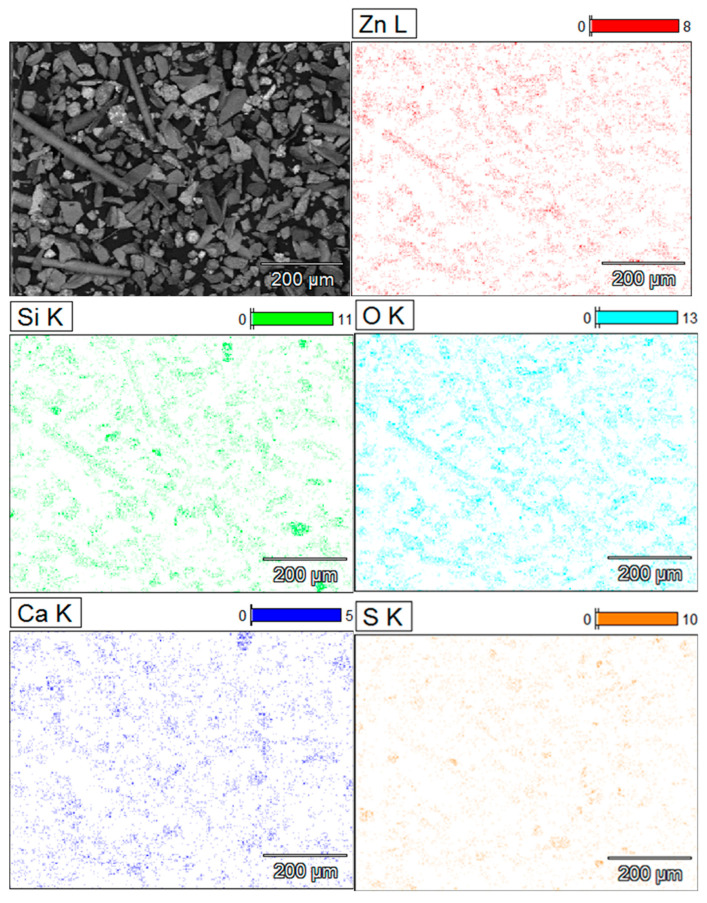
Elemental mapping of slag material showing the presence of Zinc along with silicon, and calcium in the flakes and the needle-like particles of oxide phase particles. Needles have less calcium compared to other shapes.

The results of the EDS area analysis of the particles of the slag materials infer the following:

The flaky particles (i.e., the main component of the solid slag) consist of metal oxide compounds or [(Fe, Ca, Mg, Zn, Al, Si) O]. The flake type of particles contain the highest zinc content of 11–15% (*w*/*w*) of all the particles present in the slag. The elemental mapping in Figure 8 shows the preferential location of zinc inside the regions of the flake and needle-like shape particles. The flake type of particles have the lowest contents of sulphide formers Pb and Cu. The EDS analysis showed no peaks for arsenic and germanium in these particles.

The needle-like particles are also composed of the [(Fe, Ca, Mg, Zn, Al, Si) O] compounds, however with higher silicon and lead contents, and lower calcium content than those found the flakes. An acidity index (1) of the slag is introduced to compare the chemistry of the flake shape and the needle-like particles components of the historic slag material as follows:

(1) $$i=\frac{\frac{Si\%}{28}}{\frac{Ca\%}{40}+\frac{Fe\%}{56}+\frac{Mg\%}{24}+\frac{Zn\%}{65}+\frac{Pb\%}{207}}$$

The calculation yields values of the acidity index equal to 0.5 for the flakes and 1.2 for the needle-like particles of the slag material (i.e., the acidity index of the needles is more than double that of the flakes). This difference suggests that the molten slag in the smelting furnace contained at least two different liquid phases; one main liquid phase of the composition of the flakes and a second liquid phase, in smaller quantity, of the composition as the needle-like particles. The needles phases therefore are much higher in silica-rich phases than other shapes. This second liquid might have consisted of droplets or veins that formed the elongated needles (L > 500 µm) with fine cross section diameters (D < 75 µm) upon tapping and quenching of the slag in the high-pressure water jet. The higher acid index of the needle-like shape particles indicates the tendency of formation of polymeric silicates with tridimensional structures, which results in high viscosity of the molten slag phase. The coexistence of significant numbers of the needle-like particles with the flakes in the fine granulometric size ranges may be ascribed to the separation, due to lack of miscibility, of the two phases in liquid slag inside the hearth of the furnace. Some micron and sub-micron size particles, of similar appearance to the ovoid and spherical shape phase are trapped onto the surface of the needle-like particles, not onto the flake-shaped particles. It is inferred, therefore, that the high viscosity of the liquid phase of high acidity index (needle-like) hindered the decantation of the droplets of liquid sulphide (metal) phases through the hearth zone into the matte or metal phase. 

**Figure 9:**
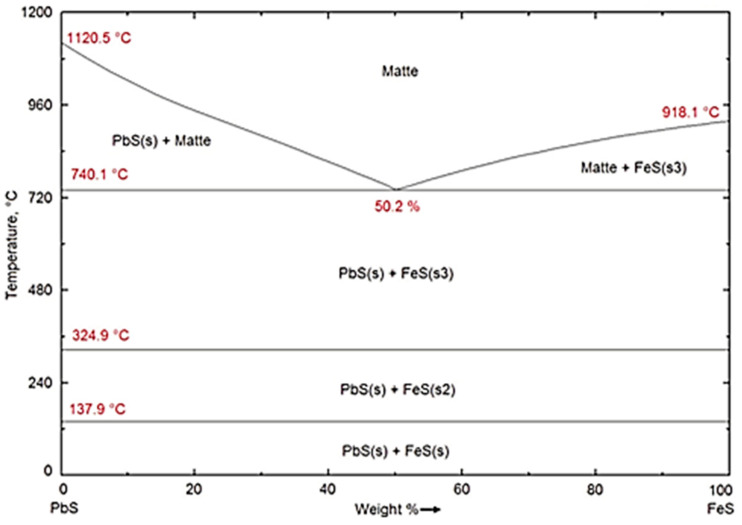
Calculated equilibrium phase diagrams showing the lack of miscibility between PbS and other metal sulphides below 600 °C.

On the other hand, the ovoid particles are distinguishable by their rough surfaces and have very low contents in oxide formers Si, Ca, Fe Mg, Al, and Zn. They rather have the highest concentrations of Cu, Fe, As, and S along with the second highest content of Pb of all the four types of particles encountered in the historic slag material. It is concluded from the results of EDS analysis in Table 3 that the ovoid particles consist of the sulphide compounds such as Cu_2_S, FeS, PbS, and As_2_S_3_ that constituted the matte product in the smelter. The wide range of variation of the four elements Cu, Fe, Pb and As is ascribed to the heterogeneity within the ovoid shape particles due to the separation of non-miscible sulphides phases upon cooling. The calculated equilibrium phase diagrams in Figure 9, show the lack of miscibility between PbS and the other sulphides such as FeS, Cu_2_S or ZnS below 600 °C. The lack of miscibility with other sulphides and metals is also exploited as a means of producing high grade mattes and Pb metal in smelting furnace.

Finally, the spherical particles with smooth surfaces have the highest Pb content of all the four types of particles, which reaches 79% (*w*/*w*) in some areas, with relatively low sulphur. The strong oxygen peak infers that these particles consist of a mixture of PbO and Pb metal formed in the melt upon the conversion reactions (2) and (3). The spherical particles have dissolved some amounts of Si, Fe, As, and Zn. The appearance of these free particles in the slag material shows that their material is not miscible either with the liquid slag (flakes and needle-like particles materials) or with the sulphide material of the ovoid shape particles.

(2) $$2PbS+3O_{2}\to 2PbO+2SO_{2}$$

(3) $$PbS+2PbO\to 3Pb+SO_{2}$$

The EDS analysis suggested the presence of arsenic and germanium was in the sulphide constituents of the ovoid shape particles and in the high Pb content spherical particles. The L_α_ X-ray energies of germanium and arsenic are close to each other at 1.188 keV and 1.282 keV, respectively. The EDS method showed little selectivity between these two elements especially in the presence of low germanium concentration and low $\frac{Ge\%}{As\%}$ as expected in this case. This method of analysis suggests that arsenic and germanium are collected in the ovoid shape particles of the sulphide phases and more in the spherical particles of the (Pb, PbO, PbS) phase. The sulphide ovoid shape particles and the lead spherical particles would contain up to 1600 ppm and 6300 ppm germanium respectively. It should however be recalled here that according to the results of particle size classification, these particles and all the slag material passing the sieve at 300 µm represent about 8% (*w*/*w*) of the total mass of slag sample received. A conservative approach based on the estimation that those particles only represent 2% (*w*/*w*) of the slag material yield a germanium content within the range 32–126 ppm in the historic smelter slag received. 

#### 3.3.3. Morphology and Composition of Coarse Size (>300 µm) Slag Particles

**Figure 10:**
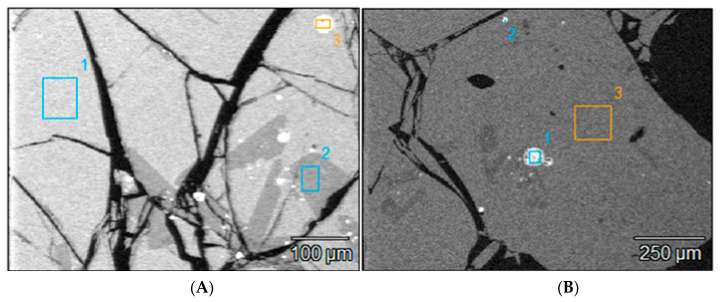
SEM BSE image showing the matrix, a secondary phase and matte fine particles areas in slag particles larger than 850 µm, (**A**) +850–3350 μm and (**B**) +3350 μm, the numbers 1, 2, 3 in the SEM micrographs represent the selected grains for SEM EDS analysis, not relevant.

The morphologies of the slag particles within the size ranges +300–600 µm, +600–850 µm, and +850–3350 µm are illustrated in Figure 4 and Figure 5. Unlike the fine particle size ranges (<300 µm) where flakes, needles, ovoid and spherical shape particles were found, the coarser particles are bulky and irregular multifaceted with multiple cracks. These coarse particles contain some amount of small particles of entrapped matte or metals as seen in the micrographs of the polished particles of sizes larger than 850 µm and 3350 µm which are shown in Figure 10. The EDS chemical analysis of the phases present in these coarse slag particles (in Table 4) indicates the presence of two slag phases and the small particles of matte or metal phases. Table 4 shows that the matrix and the secondary phases of the bulky slag particles (>300 µm) have chemical composition ranges similar to that of the flaky particles found in the fine materials (<300 µm). It therefore infers that the formation of the small flakes resulted from grinding of the molten slag by the high-velocity flow water jet, whereas the coarse bulky slag particles were formed in the low-velocity flow regions of the water jet. Pressure and flow rate distribution inside the water jet determined the granulometric distribution of the solidified slag.

The secondary phase found in the coarse slag particles has slightly higher silicon and calcium contents, but lower Pb content than the main phase of the slag matrix. The matrix and the secondary phases of the coarse particles have acidity index 0.4 and 0.7 respectively, which are close to the acidity index i = 0.5 of the flake shape particles found in the fine material of the received sample of historic smelter slag. It was noticed earlier that the needle-like shape particles only formed at high acidity index 1.2, under high silicon content (37 wt%), which leads to the polymerisation of silicate structures. These silicates have higher viscosity at the slag melting temperature.

#### 3.3.4. Optical Microscopy Analysis of the Slag Constituents

As a way of simplifying the process of identification, all phases identified are given names of natural equivalents. The process of slag formation leads also to the formation of glass and several silicate phases with specific shapes that have been identified both under the SEM and in optical microscopy. Glass is mainly silicate material that is formed when silicate melt is cooled rapidly. The petrography is better understood by realizing that in the slag development, free metal particles become droplets in the slag, and then cool down and tend to form spherical and ovoid shapes. The following sulphide phases have been identified: galena (PbS); wurtzite (ZnS); sphalerite (ZnS); chalcopyrite (CuFeS_2_); pyrrhotite (Fe_1−x_S); pyrite (FeS_2_); minor cubanite (CuFe_2_S_3_) and covellite (CuS). The silicates present include fayalite (Fe_2_SiO_4_), monticellite (CaMgSiO_4_), mellilite (Ca(Mg,Fe,Zn)Si_2_O_7_), anorthite (CaAl_2_Si_2_O_8_) and Pb-Plagioclase (PbAl_2_Si_2_O_8_). Zn, Mg, Fe, and Ca spinels and wuestite (FeO) are also present.

**Figure 11:**
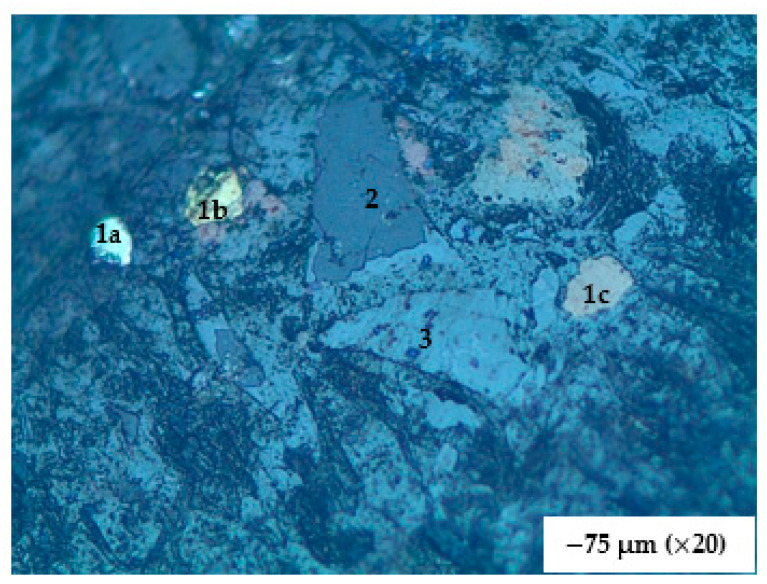
A classical occurrence of the slag material showing the different phases present. Three phases in a −75 µm size fraction. 1a (pyrite), b (chalcopyrite), and c (pyrrhotite) = metallic sulphide phases; 2 = sulphide phase (ZnS); 3 = silicate phase (spinel in slag). Length of bottom of photograph = 2 mm. The metallic phases are in the order of 0.15–0.25 mm in size.

**Figure 12:**
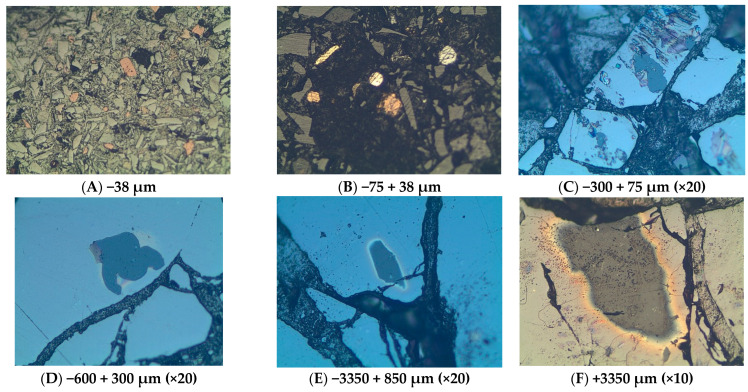
Phases (metallic, oxide and sulphide phases) within various size fractions indicated at the bottom of each photograph: (**A**) Mostly slag with minor pyrrhotite; (**B**) Grains of chalcopyrite (bright yellow) with pyrrhotite, dull yellow; (**C**) Large galena phases (PbS) with enclosed sphalerite (ZnS); (**D**,**E**) mainly galena phases enclosing low reflectance probable ZnS; (**F**) A metalloid of probably spinel with wurtzite (ZnS). Length of bottom photograph = 0.4 mm.

Six size fractions (−38, −75 + 38, −300 + 75, −600 + 300, −3350 + 850, and +3350 µm) of polished mounted samples were analyzed with the optical microscope, with results shown in Figure 11 and Figure 12. In comparing photographs from the SEM and from the optical microscopy, it is worth noting that brightness in SEM is a function of atomic number whereas in microscopy it is a function of the bonding structure. The spinels (Zn, Mg, Fe, and Ca spinels) appear to be the first ones to form in the scorification process. The metallic phases are dominant in the −38, −75 + 38, −300 + 75 µm fractions. The coarse-grained samples contain fewer grains of metallic phases. Three main phases were observed, namely the silicate phase (slag), metallic phase and the sulphide phases as shown in Figure 11. 

The analysis of Figure 12 also reveals that the metallic and sulphide phases are predominantly contained in the slag finer size fractions (−38, −75 + 38 and −300 + 75 µm) whereas the sulphide phases are also present in the coarser size fractions (300, 850 and 3350 µm).

**Figure 13:**
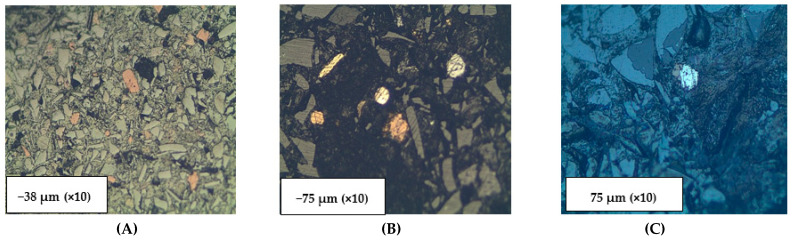
Metallic phases occurring as individual grains within the slag in the finer size fractions. In (**A**) the morphology of the slag material is observed as being of various sizes and shapes. In (**B**) (center the sulphide phases occur as spherical phases, indicating a high temperature of formation as droplets in the slag. In (**C**) (far right) is an example showing the very bright ovoid phase (sulphide); intermediate brightness, spinels and very low brightness, silicates.

The SEM EDS was used to determine the elemental composition of the phases. The metallic phase consists of elements such as copper (Cu), lead (Pb), germanium (Ge), and some zinc (Zn). Metallic phases in finer size fraction occur as interstitial grains within the slag matrix and this shows that the metals can easily be separated from the slag (Figure 13).

**Figure 14:**
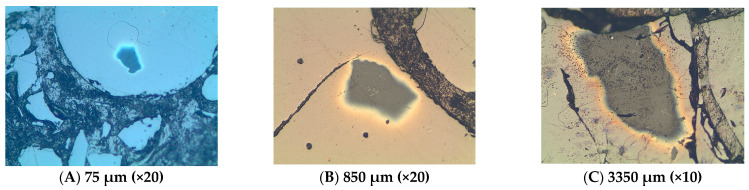
In (**A**), a nearly perfect sphere of PbS. A PbS phase occurring in (**B**) within the slag with bright edges being rimmed by CuS and ZnS. The grey phase in (**B**,**C**) has a high amount of ZnS.

The silicate phases consist mostly of olivine and pyroxenes rich in zinc whereas the sulphide phases are predominantly made up of lead sulphide (PbS) grains which are being rimmed by copper and zinc sulphides; occasionally chalcopyrite occurs as a single phase. This proposes that PbS crystallized first as droplets in the slag, followed by ZnS attaching itself, together with CuS, on the nucleated PbS phases (see Figure 14). The sulphide phase occurs within the slag matrix. 

The composition of glass was verified by the SEM EDS, and it is variable and enriched in Ca and Fe. There are several angular grains of feldspars (Figure 12 and Figure 13). These attest to the fact that the duration of melting of the historic slag in the furnace was not high enough to allow for good formation of euhedral crystals or the temperature of slag formation was not sufficiently high [45] to completely melt the furnace charge. Spinels in the slag represent the formation of oxide phases. Some of these spinels will form if oxidation conditions are not high enough in the slag, verging towards reducing conditions where sulphide droplets are stable and form minerals. The most commonly observed sulphides are galena (PbS), wurtzite and sphalerite (ZnS), pyrrhotite (Fe_1−x_ S); and various sulphides of Cu and Fe such as pyrite (FeS_2_), cubanite, covellite, and chalcocite. Wurtzite is a high temperature phase that forms at about 1020 °C [46] and contains some Fe as observed in the SEM. This suggests that temperatures of that order were at least reached in the furnace.

The important finding here is that the sulphide phases are interstitial and can be liberated from the slag. The scanning microscopy analysis of the slag material shows the presence of the free particles of matte phase and metal (ovoid and spherical) phases in material of size ranges below 300 µm. Slag particles coarser than 300 µm have trapped the matte and metal particles inside. The coarse particles of slag material have large cracks, which indicate that they are brittle and friable. The interfaces between the matte or metal particles and the slag material are incoherent with very little or no bonding strength, this results in the complete separation between them at particle sizes below 300 µm. It may therefore be necessary to mill the slag material to 300 µm to achieve the liberation of all the matte and metal particles trapped inside the coarse slag particles. 

## 4. Recommended Processing Routes for the Recovery of Valuable Metals in the Slag

A combination of mineralogical and chemical methods indicated that the historic slag from Tsumeb smelter contains extractable metals such as copper, zinc, lead and arsenic whose average compositions are 0.35% Cu, 5.5% Zn, 3% Pb, and 0.2% As, respectively. Ge is a trace element, with its presence only observed using the SEM. Germanium as a strategic metal is widely used in advanced technology applications such as thermal solar panels and optic fibres. Germanium is currently extracted from zinc and lead sulphide ores [47,48] or coal deposits as well as metallurgical residues from the processing of the sulphide ores and coals (e.g., smelting flue dust and coal fly ashes) by volatization concentration from lignite coal [49]. Typical concentrations of Ge in these resources range from 30 to 200 ppm [50,51,52]. The estimated content range of 32–126 ppm germanium in the historic Tsumeb slag is well comparable to that in resources from which it is obtained as a by-product of metal sulphide treatment worldwide by hydrometallurgy and pyrometallurgy processes [40,49,51]. The world’s germanium production was only 300 metric tons coming from China (about 60%), Canada, Finland, Russia, and the United States [53]. The demand forecast of germanium estimates an annual growth rate of 3.5% during the period time 2023–2030. Germanium ingots trade price in London was about U$1,300,000/ton in July 2023, which is about 500 times the prices of zinc and lead, which were priced at about U$2300 and U$2100/ton respectively, and copper traded at U$8300/ton during the same period. The higher price justifies the extraction of germanium from low-grade resources. The simultaneous extraction of zinc, lead, copper, and germanium from the Tsumeb historic slag can hence be justified economically. The suggested processing routes for the metals in the Tsumeb slag are discussed below.

- Copper, lead and arsenic

Energy dispersive of X-ray spectroscopy analysis shows that copper, arsenic and most of lead are contained in round shaped sulphide particles which can easily be detached from the slag matrix. These particles can be liberated by low-energy milling of the slag to below 106 μm. Flotation, with xanthate collector, can be applied to concentrate these sulphide minerals into a feed recycle to the smelter [8]. Alternatively, the sulphide concentrate can be roasted to produce the calcine which can be advanced to the leaching tank to recover copper [38].

b. Zinc and lead

The SEM EDS analysis shows that most of zinc and fraction of lead are found in dissolved form in the slag matrix material. Recovery of zinc and lead from the complex silicate compound requires chemical reactions such as reduction with solid coal and fuming at temperature above the melting temperature of the slag [54]. Alternatively lead and zinc can be leached from the slag matrix in alkaline medium. This is possible since Pb and Zn can both form complexes with hydroxyl ions (OH^−^) [55]. Alkaline leaching presents the advantage of lower dissolution of iron and contamination of the leaching solution [56]. It also avoids the risk of formation of gel due to the collapse of silicate structures in acidic medium [57]. Purification methods such as cementation can then be applied to recover Pb and Zn metals from the pregnant leach solution.

c. Germanium

SEM EDS analysis have revealed the presence of germanium in the sulphide particles with lead content. Germanium can be recovered simultaneously with lead, copper and zinc sulphides. Germanium can also be recovered by acid leaching of the white alloy produced by direct reduction of the slag with solid coal, followed by solvent extraction and precipitation. Such process is implemented in the Democratic Republic of Congo for the simultaneous recovery of copper, cobalt, zinc, and germanium from copper matte smelting slag [22,33]. 

## 5. Conclusions

The depletion of the ore reserves in the world necessitates the search for secondary sources such as slags for the continued production of metals. Environmental and socio-economic impacts of treating such slags and further recovering valuable metals from them cannot be overlooked. The smelter in Namibia accumulated a significant amount of blast furnace slag during its operating life which can be traced back to 1900’s. A representative slag sample was prepared for mineralogical characterization using a combination of analytical and mineralogical techniques (AAS, XRF, ICP OES, SEM EDS and optical microscopy). The chemical analyses showed that the metal values contained in the slag were mainly copper, lead, and zinc whose average contents were approximately up to 0.4% Cu, 4% Pb and 7% Zn. About 11% Fe was also contained in the slag. Germanium is a trace element, with its concentration estimated from the SEM EDS analysis to be in the range of 32–126 ppm. Optical and scanning electron microscopy have shown the mineral phases for both metals and slag. This information is useful in developing the extraction methods for various metals in the slag. 

The granulometric classification shows high volume fractions of sulphide particles present in the slag material of the size ranges smaller than 300 µm, and are readily separable from the slag material. The presence of liberated sulphide particles shows that there is no interface bonding between the sulphide phases and the slag matrix, thus mechanical separation between these phases is feasible.

Most sulphide and metal particles are trapped inside the slag particles larger than 300 µm, which represent about 90% of the slag material. The large cracks observed in the SEM and optical images suggest that these coarse slag particles are brittle and friable. Low energy milling will therefore suffice to fracture the large slag particles as a means to liberate the trapped sulphide particles. 

Based on the elemental analysis and mineralogical characterisation work undertaken, it is recommended that metallurgical test work be carried out to determine an economically viable and safe process route for recovery of valuable metals contained in the slag. The target metals would include Zn, Pb, Cu, Ge, Mo, and Ga. The metallurgical test work proposed includes pre-concentration of valuable minerals using gravity separation of the sulphide particles from the slag matrix material. Zinc can be extracted by coal reduction and fuming or by leaching. Germanium can be extracted by leaching the white alloy obtained by direct reduction of the slag with coal or from the concentrate of sulphide minerals.

## Figures and Tables

**Table 1:** Compositions of copper smelter slags in wt%, Refs. [7,22,25,26].

Copper Smelting Method	FeO + Fe_3_O_4_	SiO_2_	CaO	MgO	Al_2_O_3_	Cu	S	Co	ZnO	Au + Ge + Ag
Water jacket furnace (DR Congo)	29	31	10	5	7	1.4	0.6	2	10	10 g/ton
DC electric furnace (DR Congo)	21	38	12	11	6	0.3	0.1	0.5	4	-
Mitsubishi	54	32	7	-	4	2	0.6	-	-	-
ISASMELT method	49	33	2.3	2	0.2	1	2.8	-	-	-
Converter slag	76	22	2	1	8	2	4	-	-	-
Inco flash smelter	<60	<33	1.7	1.6	4.7	0.9	1.1	-	-	-
Vanukov smelting	<58	<24	2	1.5	3	2.5	0.6	-	-	-
Airtight blast furnace	<43	<35	<12	4	8	1.4	0.3	-	-	-
Flash smelting	<59	<33	<10	2	7	0.25	0.6	-	-	-

**Table 2:** Elemental compositions of the composite historic smelter slag samples and assay-by size (+3.35 mm and −75 µm).

Sample Type	Element (% *w*/*w*)											
	Fe	Si	Ca	Mg	Al	Mn	Cu	Zn	Pb	As	Mo	Precious Metals
Composite samples	9–11	13–19	11	4	2	0.1	0.3–0.4	4–7	2–4	0.2	0.25	
Sieved at +3350 μm	10		11			0.13	0.3	3.2	3.2	0.2	0.2	
Sieved at −75 μm	11		8			0.12	1.2	3.1	3.5	0.8	0.2	

**Table 3:** EDS semi-quantitative area analysis composition ranges in % (*w*/*w*) of particles in received slag material passing the 300 µm sieves. (The oxygen and sulphur contents are only indicative of the oxide and sulfide phases present).

Element	Flakes	Needles	Ovoid	Spheres
Si	13–19	14–37	<0.4	0.3–9.0
Ca	12–20	<9.0	-	<1.8
Fe	4–21	12–20	1.0–39	<9.0
Zn	11–15	8.7–17	0.3–1.4	1.7–3.0
Pb	0.7–2.6	3.4–7.0	15–39	48–79
Cu	0.0–1.2	0.0–0.93	12–44	3.7–4.4
As	-	-	2.5–30	2.5–15
Ge	-	-	<0.16	<0.63
Mg	3.8–5.3	<5.0	-	<0.07
Al	1.1–2.8	1.9–2.8	-	-
S	<0.6	<0.4	1.3–55	<4.0
O	29–37	27–34	<7.0	9.0–20

**Table 4:** EDS semi-quantitative area analysis composition ranges in % (*w*/*w*) of the phases observed in coarse slag particles (+300–3350 µm). The oxygen and sulphur contents are only indicative of the oxide or sulphide compounds present.

Element	Slag Matrix	Secondary Phase of the Slag	Trapped Matte/Metal Particles
Si	12–13	19	1.2–8.7
Ca	12–16	21	0.5–1.8
Fe	19–23	3	4.3–8.6
Zn	13–17	13	2.3–3.0
Pb	<6.9	<0.7	48–59
Cu	-	-	15–16
As	-	-	2.7–3.2
Ge	-	-	0–0.63
Mg	3.2–4.1	4.7	-
Al	2.0–2.1	1.2	0.2–0.4
S	<0.3	-	0.4–0.9
O	25–29	38	9.7–12

## Data Availability

Data is contained within the article.

## Appendix A. Assay-by Size

**Table A1:** Assay by size from AAS.

Elements	Elemental Composition (% *w*/*w*)				
	+3350 µm	−3350 + 850 µm	−850 + 300 µm	−300 + 75 µm	−75 µm
Si					
Ca	11.07	11.81		10.81	8.34
Mn					
Fe	9.01	11.97		10.7	11.05
Cd					
Ni					
Cu	0.33	0.33		0.51	1.51
Zn	0.8	0.86		0.86	1.05
As					
Mg					
Co					
Pb	3.33	3.97		4.37	5.53
Ag					
Ge					
Mo					
Other (%)	75.46	71.06		72.75	72.52

**Table A2:** Assay by size from ICP.

Elements	Elemental Composition (% *w*/*w*)				
	+3350 µm	−3350 + 850 µm	−850 + 300 µm	−300 + 75 µm	−75 µm
Si					
Ca					
Mn	0.12	0.14	0.15	0.13	0.09
Fe	10.45	14.29	17.81	14.53	9.75
Cd	0.01	0.01	0.01	0.02	0.05
Ni	0.001	0.001	0.002	0.001	0.001
Cu	0.28	0.33	0.35	0.45	1.08
Zn	3.08	3.12	3.16	2.98	2.1
As	0.23	0.3	0.32	0.38	0.71
Mg	2.45	2.66	2.9	2.67	0
Co	0.01	0.012	0.012	0.011	0.008
Pb	2.03	2.23	2.28	2.23	2.04
Ag	0.0013	nd	0.004	nd	nd
Ge	nd	nd	nd	nd	nd
Mo					
Other (%)	81.3377	76.907	73.002	76.598	84.171

**Table A3:** Assay by size from XRF.

Elements	Elemental Composition (% *w*/*w*)				
	+3350 µm	−3350 + 850 µm	−850 + 300 µm	−300 + 75 µm	−75 µm
Si					
Ca					
Mn	0.13	0.13	0.08	0.15	0.14
Fe	10.02	8.96	5.35	8.22	11.91
Cd					
Ni					
Cu	0.26	0.21	0.11	0.2	1.06
Zn	7.13	5.7	2.93	4.72	7.37
As	0.18	0.14	0.08	0.2	0.88
Mg					
Co					
Pb	2	1.55	0.74	1.07	2.92
Ag					
Ge					
Mo	0.19	0.12	0.06	0.11	0.19
Other (%)	80.09	83.19	90.65	85.33	75.53
